# Obituary

**Published:** 1908-12-12

**Authors:** 


### OBITUARY.

The death occurred on December 5, at his residence, 135 Harley Street, of Charles Edward Beevor, M.D., F.R.C.P., physician to the National Hospital for the Paralysed and Epileptic. Dr. Beevor, who was born in London in 1854, was the eldest son of the late Mr. Charles Beevor, F.R.C.S., and was educated at Blackheath Proprietary School and University College, London. He also studied at Vienna, Leipzig, and Berlin. He qualified in 1878, obtained his M.D. at London University in 1881, and the Fellowship of the Royal College of Physicians in 1888. He was Croonian Lecturer at the Royal College of Physicians in 1903, and Lettsomian Lecturer at the Medical Society of London in 1907. In 1907 he was president of the Neurological Society. In addition to his appointment at the National Hospital he was consulting physician to the National Orthopædic Hospital, and physician to the Great Northern Central Hospital. Dr. Beevor was the author of many important contributions to the study of neurology, including his volume entitled "Diseases of the Nervous System."

Dr. Andrew J. McCosh, who was seriously injured on November 28 in a carriage accident in New York, died on the night of December 2. After the accident Dr. McCosh was removed to the Presbyterian Hospital, suffering from fractured base of the skull. We learn from the *Times* that Dr. McCosh, who was born in Belfast in 1858, was the son of Dr. James McCosh, formerly professor of logic at Queen's College, Belfast, and afterwards president of Princeton College. He himself took his M.A. degree at Princeton, and proceeded to the College of Physicians and Surgeons at New York, where he became M.D. in 1880. He afterwards studied at the University of Vienna. Dr. McCosh's practice at New York extended over twenty-five years. He had been surgeon to the Presbyterian Hospital since 1889, and was professor of clinical surgery at Columbia University, which three years ago conferred on him the LL.D. degree. Dr. McCosh's last eminent patient was Ex-President Cleveland.

Dr. Francis Pritchard Davies, who was for many years superintendent and senior assistant medical officer at the Kent County Asylum, Maidstone, has recently died at the age of sixty-four. He was formerly Assistant Medical Officer to the Criminal Lunatic Asylum, Broadmoor, and he had held the posts of President of the Royal Medical Society of Edinburgh and medical officer to the Birmingham and Midlands Free Hospital for Sick Children. Dr. Davies qualified as a member of the Royal College of Surgeons in 1869 and obtained the degree of M.D. at Edinburgh University in 1879.

Professor Eduard G. von Rindfleisch, the eminent pathologist, who died on December 5 at Würzburg, at the age of seventy-two, was a pupil of the late Professor Virchow and eventually succeeded that master in the Chair of Pathology at the University of Würzburg. After studying at Heidelberg and Halle, he proceeded, in 1860, to Berlin, where he first came under the influence of Virchow's teaching. He was successively assistant at the Physiological Institute at Breslau, professor of Pathology at Zürich, and professor in the University of Bonn. His "Manual of the Doctrine of Cellular Pathology" appeared in 1867, and in 1883 he published "Elements of Pathology."

